# Physical Activity and Bone Health in Schoolchildren: The Mediating Role of Fitness and Body Fat

**DOI:** 10.1371/journal.pone.0123797

**Published:** 2015-04-27

**Authors:** Ana Torres-Costoso, Luis Gracia-Marco, Mairena Sánchez-López, Blanca Notario-Pacheco, Natalia Arias-Palencia, Vicente Martínez-Vizcaíno

**Affiliations:** 1 School of Nursing and Physiotherapy, University of Castilla La Mancha, Toledo, Spain; 2 CHERC (Children’s Health and Exercise Research Centre) Sport and Health Sciences, University of Exeter, Exeter, United Kingdom; 3 GENUD “Growth, Exercise, Nutrition and Development” Research Group, University of Zaragoza, Zaragoza, Spain; 4 Social and Health Care Research Center, University of Castilla La-Mancha, Cuenca, Spain; 5 Faculty of Education, University of Castilla La Mancha, Ciudad Real, Spain; 6 Facultad de Ciencias de la Salud, Universidad Autónoma de Chile, Talca, Chile; Medical University of South Carolina, UNITED STATES

## Abstract

**Background:**

The relationship between physical activity (PA) and bone health is well known, although the role of percent body fat (%BF) and fitness as confounders or mediators in this relationship remains uncertain.

**Objective:**

To examine whether the association between PA and bone mineral content (BMC) is mediated by %BF and cardiorespiratory fitness (CRF).

**Methods:**

In this cross sectional study, BMC, total %BF (by DXA), vigorous PA (VPA), CRF, age and height were measured in 132 schoolchildren (62 boys, aged 8–11 years). ANCOVA was used to test differences in BMC by %BF, CRF and VPA, controlling for different sets of confounders. Simple mediation analyses and serial multiple mediation analyses were fitted to examine whether the relationship between PA and BMC is mediated by %BF and fitness.

**Results:**

Children with high %BF had higher total body BMC than their peers after controlling for all sets of confounders. Children with good CRF or VPA had significantly less total body BMC after controlling for age and sex but in children with good CRF this inverse relation disappeared after adjusting by %BF. %BF and CRF both act as a full mediator in the association between VPA and BMC, after inclusion of the potential confounders in the models.

**Conclusion:**

Fitness and %BF seem to have a mediator role on the relationship between physical activity and bone mass.

## Introduction

Osteoporosis remains a major public problem due to its association with fragility fractures [1]. Peak bone mass acquired through bone mineral accrual during childhood and adolescence may be a key determinant of bone health and future fracture risk during adulthood [2].

Childhood and adolescence are crucial periods for the development of the skeleton. Cardiorespiratory fitness (CRF) [3,4,5] and physical activity (PA) [6,7] have been positively related with bone outcomes. A positive relationship between CRF and total body BMC has been shown in adolescents [3] which seems to be linked to muscle strength [8] and lean mass, with the former stablished as a strong predictor of bone mass in paediatric populations [9,10]. Moderate or vigorous PA, but particularly vigorous PA (VPA), weight-bearing PA and sports inducing higher than normal bone loads are crucial for strengthening the bones [11,12].

Physically active youngsters have higher levels of lean mass [13]. In addition, a positive association between fat mass and bone outcomes has also been observed [14,15].This controversy may be explained by the mechanostat theory [16], that recognizes that muscular strains stimulates bone modeling and mineralization, and as consequence supports that both physical activity and excessive body mass may serve as mechanical stimuli to the strains required to initiate bone adaptations. Overweight and obese children and adolescents have higher bone mineral content (BMC) and bone mineral density (BMD) than their normal-weight peers [10,14], and this association might to be explained by their higher levels of fat mass which may be a consequence of the extra weight they have to carry in their daily life activities. However, studies in children are scarce and the results controversial have been described. While Hrafnkelsson et al. [17], reported a positive association between fat mass and BMC and total bone area in children, Cole et al. [18] observed a negative association with volumetric bone density independent of lean mass, although also reported a positive association with bone size.

Despite the fact that the relationship between VPA, fitness and %BF with bone outcomes in young populations has been extensively described, no studies have jointly examined the associations of these predictors with bone outcomes. Furthermore, most published studies have been conducted using statistical multivariate procedures (ANCOVA, multiple linear regression or logistic regression) in order to adjust for potential confounders, but these statistical procedures do not report the partial and semi-partial correlations and fail to distinguish between confounding and intermediate variables.

The present study aims to disentangle, using mediation analysis statistical procedures, whether the relationship between vigorous physical activity and bone mineral content is mediated by fitness and percent body fat.

## Materials and Methods

### Subjects and Study Design

This study was a cross-sectional analysis of baseline data from a cluster randomised trial aiming to assess the effectiveness of a PA program for the prevention of excess weight in schoolchildren [19]. This study included 1592 schoolchildren, aged 8—to -11 years, from 20 public primary schools in the Province of Cuenca, Spain, during 2010–2011. For this report, we used data from a sub-sample of 132 Caucasian children (62 boys) in which BMC and BMD (by DXA) were measured. The children included in the data analysis for this study did not differ in age, sex or parental socioeconomic status from the whole sample of children participating in the trial.

The Clinical Research Ethics Committee of the Virgen de la Luz Hospital in Cuenca approved the study protocol. After obtaining the approval of the Director and Board of Governors (Consejo Escolar) of each school, a letter was sent to all parents of children in the fourth or fifth grade inviting them to a meeting, at which the study objectives were outlined and written approval for their children's participation was obtained. Informative talks, in which the school children were asked to collaborate, were then held class-by-class.

### Anthropometrics Measurements

The participating children, wearing light clothing, were weighed twice to the nearest 0.1kg with a portable electronic scale (SECA Model 861; Vogel & Halke, Hamburg, Germany). Height was measured twice to the nearest 0.1cm using a wall-mounted stadiometer, (SECA 222, Vogel & Halke, Hamburg, Germany) with the children standing straight against the wall without shoes to align the spine with the stadiometer. The head was positioned with the chin parallel to the floor. The mean of the two measurements of weight and height was used to calculate BMI as being weight in kilograms divided by the square of the height in meters (kg/m^2^).

### Analysis of Body Composition

Children were scanned in supine position and at high resolution using dual energy x-ray absorptiometry (DXA) (Lunar iDXA, GE Medical Systems Lunar, Madison, WI 53718 USA). The analyses were performed using enCORE 2008 software, version 12.30.008. The DXA equipment accuracy was checked on a daily basis before each scanning session, using the GE Lunar calibration phantom as recommended by the manufacturer. The same operator performed all scans and body composition analysis. BMC (g), BMD (g/cm2), total percent body fat (%BF) and lean mass (g) [body mass-(fat mass + bone mass)] were obtained for each individual from a whole body scan. BMD was calculated using the formula BMD = BMC/area. For the purpose of the present study, total %BF was categorized as follows: low (1st quartile), medium (2nd and 3rd quartiles) and, high (4th quartile).

### Cardiorespiratory Fitness

All participants performed a general warm up before fitness testing guided by a member of the research team lasting four minutes. Cardiorespiratory fitness (CRF) was assessed by the 20 m shuttle run test [20]. The test was explained beforehand to all participants followed by a demonstration. In addition, a researcher ran with them the first shuttles to make sure all participants knew what they had to do.Participants were required to run back and forth between two lines 20m apart, while keeping pace with audio signals emitted from a pre-recorded compact disc. The initial speed was 8.5 km/h, which was increased by 0.5 km^-1^ every minute [20]. The schoolchildren were encouraged to keep running for as long as possible through the course of the test. The test for each child was finished when that child stopped due to fatigue or when he/she did not reach the line within the time of the audio signal on two consecutive occasions. The last half stage completed was recorded as an indicator of his or her CRF. Finally, CRF was categorized as poor (first quartile), satisfactory (second and third quartiles), and good (fourth quartile) [21].

### Physical Activity

Physical activity was measured using the MTI accelerometer model 7164 (ActiGraph, Shalimar, FL, USA). The children were instructed to place the monitor on the right hip for seven consecutive days. They were also instructed to wear the accelerometer at all times, including during the night, and only to remove it during water-based activities, i.e. swimming or having a shower or bath. The accelerometer was set to record PA data every minute (60s epoch). The KineSoft (v. 3.3.2.0 software) was used to analyse the data. Sequences of 10 or more consecutive zero counts were considered to be non-wearing time and were excluded from the analyses. A minimum of four days’ recording (including at least one weekend day) with 10h of recorded time per day was set as the inclusion criteria. Time spent (min/day) at different intensities was calculated using the cut-off points of Evenson et al.[22]: sedentary time (0–100 cpm), light PA (101–2295 cpm), moderate PA (2296–4011 cpm) and VPA (> 4012 cpm). Moderate to vigorous physical activity (MVPA) was calculated as the sum of time spent in moderate and vigorous PA. Finally, VPA was categorized as poor (first quartile), satisfactory (second and third quartiles), and good (fourth quartile).

### Statistical Analysis

Both statistical (Kolmogorov Smirnov test) and graphical methods (normal probability plots) were used to examine the fit to a normal distribution for each continuous variable. All of them fitted acceptably to a normal distribution. Participants’ characteristics were described as mean ± standard deviation (SD).

Partial correlation coefficients controlling for age were calculated to assess the relationships among variables. ANCOVA models were estimated to test mean differences in total body BMC by total %BF, total lean mass, CRF and VPA categories, using total body BMC as a dependent variable. When the categories of total %BF or total lean mass and sex were entered as fixed factors, age, vigorous physical activity and cardiorespiratory fitness were used as covariates. When the categories of CRF and sex were entered as fixed factors, age, VPA and total %BF or total lean mass were the covariates. Finally, when the categories of vigorous physical activity and sex were entered as fixed factors, age, CRF and total %BF or total lean mass were the covariates.

Mediation analyses were conducted to examine whether the association between PA and bone mass was mediated by %BF and fitness. Two different approaches were used in the mediation analyses:

a. Simple mediation analysis. Linear regression analyses were performed to test for the potential mediating effect of CRF and total %BF in the association between VPA and total body BMC following the criteria outlined by Baron and Kenny [23]: 1) the independent variable must be significantly related to the mediator; 2) the independent variable must be significantly related to the dependent variable; 3) the mediator must be significantly related to the dependent variable; and 4) the association between the independent and dependent variable must be attenuated when the mediator is included in the regression model.

In addition, we tested mediation using the steps outlined by Sobel [24]: Firstly, we estimated the attenuation or indirect effect (i.e., the effect of the independent variable on the mediator from the first regression model, multiplied by the effect of the mediator on the dependent variable obtained from the third regression model). Secondly, we divided the indirect effect by its standard error and performed a z test under the null hypothesis that the indirect effect is equal to zero.

**Fig 1:**
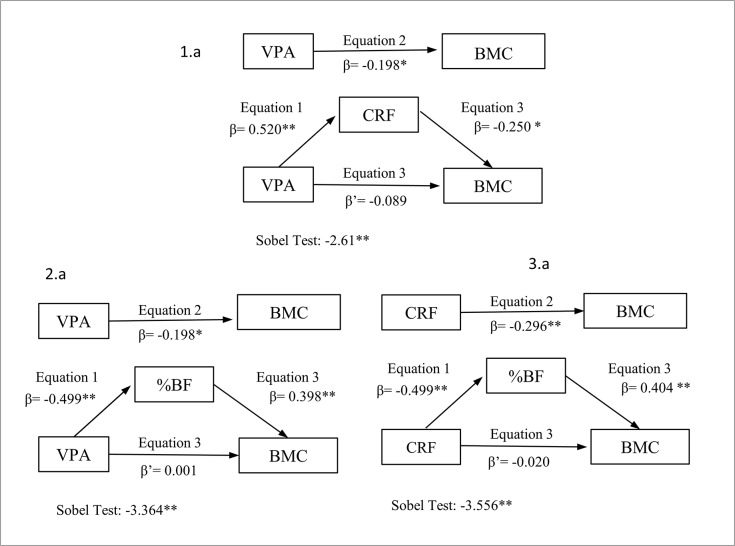
Simple mediation models of the relationship of vigorous physical activity (VPA) and cardiorespiratory fitness (CRF) with bone mineral content (BMC), using percent body fat (% BF) or CRF as mediators, controlling for age. **p< 0.01 *p< 0.05

**Fig 2:**
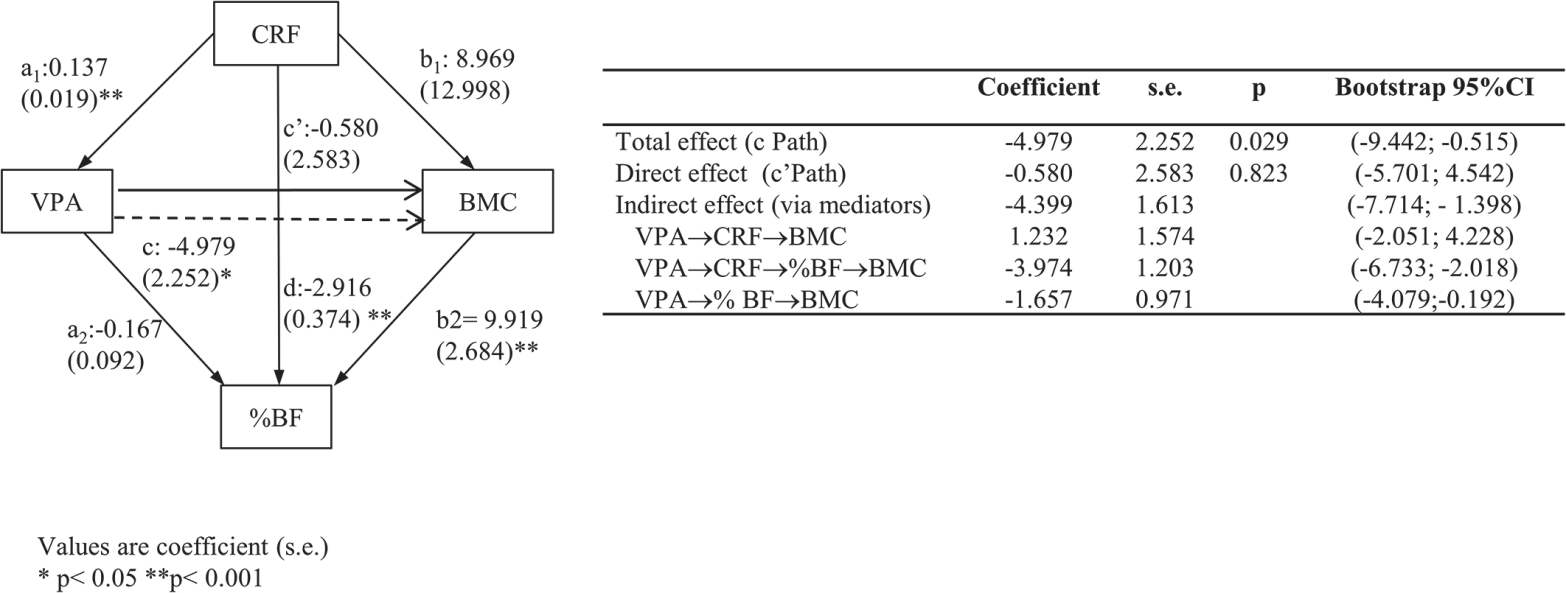
Multiple mediation analyses of the association between vigorous physical activity (VPA) and total body bone mineral content (BMC), with cardiorespiratory fitness (CRF) and percent body fat (% BF) as mediators, controlling for age.

Thus, the first regression model examined whether the association between VPA and total body BMC was mediated by CRF. The second regression model examined whether the association between VPA and total body BMC was mediated by %BF. The third regression model examined whether the association between CRF and total body BMC was mediated by %BF. All regression models were adjusted for age (Fig 1).

b. Serial multiple mediation analysis. This was a more complex serial multiple mediator model specifying the sequence of mediation as follows: VPA→CRF→ %BF→ BMC (Fig 2).

Both simple and serial mediation models were estimated by using the PROCESS macro for SPSS. This macro used bootstrapping methods as recommended by Preacher and Hayes [25] for testing mediation hypotheses (we used a resample procedure of 10,000 bootstrap samples). To estimate the serial mediation models the order of variables must have been previously pre-determined.

Mediation analyses were conducted to examine whether the association between VPA or CRF and bone mass was mediated by lean mass. In addition, all the analyses were conducted using total body BMD as a dependent variable.

Statistical analyses were performed with SPSS-IBM (Software, v.19.0 SPSS Inc., Chicago, IL, USA), and the level of significance was set at α = 0.05.

## Results

Table 1 shows the descriptive characteristics (mean ± SD) of the study sample. None of the variables significantly differed by sex except for CRF, MVPA and LM, in which boys had higher values than girls (p<0.05).

**Table 1:** Descriptive characteristics of the study sample.

	All (132)	Boys (62)	Girls (70)	p
Age (years)	9.43±0.72	9.38±0.78	9.47±0.67	0.484
Height (cm)	140.16±6.63	139.46±6.85	140.74±6.42	0.272
BMI (Kg/m^2^)	18.84± 3.76	19.43±4.39	18.35±3.07	0.112
CRF (mean stage)	3.57±1.83	4.02±2.13	3.21±1.45	**0.014**
Maximum stage reached (n, %)				
≤ 3	50, 37.6%	22, 16.7%	28, 21.2%	
3 ≥ x < 6	63, 47.4%	22, 16.7%	41, 31.1%	
≥ 6	19, 14.4%	16, 12,1%	3, 2.3%	
Total body BMC (g)	1357.49±195.46	1369.63±202.90	1343.91±188.7	0.453
Percentage body fat (%)	12.79±6.02	12.95±7.14	12.61±4.96	0.748
Total lean mass (Kg)	26.11±4.00	26.84±4.44	25.44±3.49	**0.048**
MVPA (min day^-1^)	46.88±23.57	55.75±25.27	39.61±19.44	**<0.001**
VPA (min day^-1^)	9.28±7.37	10.65±8.52	8.15±6.12	0.086

Values are mean ± SD.

CRF cardiorespiratory fitness; BMI body mass index; BMC bone mineral content; MVPA moderate-to-vigorous physical activity; VPA vigorous physical activity.

Table 2 shows age-adjusted partial correlations among total %BF, total LM, CRF, MVPA, VPA and total body BMC. A positive correlation was found between total body BMC and total %BF (r = 0.417) and total LM (r = 0.866) while negative correlations were found between total body BMC and CRF (r = -0.257) and VPA (r = -0.208).

**Table 2:** Partial correlation coefficients among total body bone mineral content, physical activity, total percent body fat, total lean mass and cardiorespiratory fitness in children, controlling for age.

	Total body BMC	% BF	TLM	CRF	MVPA	VPA
Total body BMC	-					
% BF	0.417**	-				
TLM	0.866**	0.425**	-			
CRF	-0.257*	-0.712**	-0.254*	-		
MVPA	-0.110	-0.391*	-0.092	0.464**	-	
VPA	-0.208*	-0.500**	-0.202*	0.560**	0.803**	-

BMC bone mineral content; Total % BF Total percent body fat; TLM Total lean mass; CRF cardiorespiratory fitness; MVPA moderate to vigorous physical activity; VPA vigorous physical activity.

* p< 0.05;

**p< 0.001.

Table 3 shows the mean-adjusted differences in total BMC by total %BF, CRF and VPA categories, using total body BMC as a dependent variable. Children with high total %BF had higher total body BMC than their peers after controlling for all sets of confounders in all models. Moreover, children with better CRF had significantly lower total body BMC than those with worse levels after controlling for age and sex (model 1), but these significant differences disappeared when controlling for VPA (model 2) and total %BF (model 3). Finally, children that spent more time doing VPA had significantly lower total body BMC than those with worse levels after controlling for age and sex (model 1), but these significant differences disappeared when controlling for CRF (model 2) and total %BF (model 3).

**Table 3:** ANCOVA models comparing means of total body bone mineral content (BMC) by total percent body fat (%BF), cardiorespiratory fitness (CRF) and vigorous physical activity (VPA) categories in children.

Total body BMC (g)												
	Total body fat (%)				Cardiorespiratory fitness				Vigorous physical activity			
	Low	Medium	High		Poor	Satisfactory	Good		Poor	Satisfactory	Good	
	n = 33	n = 66	n = 33	p	n = 38	n = 60	n = 24	p	n = 32	n = 55	n = 25	p
Model 1	1253.01±26.66	1361.94±21.92	1461.04±29.62^a^	**<0.001**	1451.20±29.11^a^ ^,^ ^b^	1326.04±24.23	1302.92±33.35	**0.001**	1405.32±32.59^a^	1330.60±23.62	1286.91±35.85	**0.046**
Model 2	1255.52±34.57	1347.95±24.44	1438.75±34.88^b^ ^,^ ^c^	**0.003**	1399.63±33.81	1323.72±27.07	1306.38±37.88	0.147	1386.20±33.08	1325.92±23.24	1323.68±38.78	0.301
Model 3	1259.44±38.55	1347.86±24.56	1434.46±39.52 ^c^	**0.025**	1321.81±38.25	1332.35±25.66	1369.76±39.66	0.695	1362.57±32.23	1329.19±22.16	1346.40±37.50	0.675

Covariates for %BF: Model 1(age and sex); Model 2 (Model 1+ VPA); Model 3: (Model 2+CRF). Superscript letter indicates statistical significance (≤ 0.050) for post hoc hypothesis test determinates by using the Bonferroni correction for multiple comparisons:

^a^ High >Medium> Low;

^b^ High> Medium;

^c^ High> Low.

Covariates for CRF: Model 1(age and sex); Model 2 (Model 1+ VPA); Model 3: (Model 2+%BF) Superscript letter indicates statistical significance (≤ 0.050) for post hoc hypothesis test determinates by using the Bonferroni correction for multiple comparisons:

^a^ Poor> Satisfactory;

^b^ Poor> Good.

Covariates for VPA: Model 1(age and sex); Model 2 (Model 1+ CRF); Model 3: (Model 2+%BF). Superscript letter indicates statistical significance (≤ 0.050) for post hoc hypothesis test determinates by using the Bonferroni correction for multiple comparisons:

^a^ Poor> Good.

### Simple mediation analysis

We tested the mediator role of CRF (Fig 1A) and total %BF (Fig 1B) in the relationship between VPA and total body BMC and also the mediator role of total %BF in the relationship between CRF and total body BMC (Fig 1C). Both CRF and total %BF acted as total mediators of the relationship between VPA and total BMC; thus, when either CRF or total %BF were considered, the relationship between VPA and total BMC became statistically not significant. In the same way, total %BF acted as a total mediator of the relationship between CRF and total body BMC.

The percentage of total effect mediated by CRF was 16.4% (Fig 1A, z = -2.61; p≤0.001); the percentage of the total effect mediated by %BF was from 25.6% to 28.6% (Fig 1B, z = -3.36; Fig 1C z = -3.56; p≤0.001).

### Multiple mediation analysis

When multiple serial bootstrapped mediation models were estimated (Fig 2) entering CRF and total %BF, and controlling for age, the relationship between VPA and total body BMC did not remain significant (p = 0.823). Thus the model was fully mediated. The significant paths in the model mediating this relationship between VPA and BMC were the following: VPA→%BF→BMC (95% CI: -4.079; -0.192) and VPA→CRF→%BF→BMC (95% CI: -6.733; -2.018). Indirect effect through the path VPA→CRF→BMC was not significant.

When we tested the independent association of lean mass with BMC and the mediator role of lean mass in the relationship between VPA and BMC similar results were found (please see S1 Table and S1 and S2 Figs). Additionally, further analyses were performed by using total body BMD as a dependent variable and similar results were obtained (data not shown).

## Discussion

The present study is, to our knowledge, the first to disentangle the role of %BF and CRF in the relationship between VPA and BMC in schoolchildren using multiple mediation analysis. Our data confirmed the independent association of total %BF, CRF and VPA with total body BMC. Children with higher %BF and with poor VPA and CRF exhibited a greater total body BMC than their peers in other categories of %BF, VPA or fitness. In addition, our data showed that the CRF and %BF act as total mediators of the relationship between VPA and total BMC and, in the same way, %BF acts as a total mediator of the relationship between CRF and total body BMC. Finally, a serial mediation model displayed that the relationship between VPA and total body BMC was fully mediated by a set of variables consisting of CRF and %BF, controlling for age. The complex relationships of this serial model evidenced that: i) changes in VPA influence positively the CRF levels; ii) those CRF levels influence negatively the %BF; and iii) %BF was positively associated with total body BMC.

### Percent Body Fat and BMC

Body weight has been identified as one of the main determinants of BMC gain [26,27] and can positively influence weight-bearing bones [14,28]. Overweight children have not only more fat mass, but also more lean mass [29], which is an excellent indicator of the mechanical stimulation of bone [30]. The mechanostat theory [16] might explain these findings because bones adapt, not only to static forces (from excess of weight), but also to the dynamic forces caused by muscle contractions; however it has been observed that both lean and fat mass were associated with BMC and bone area [17]. In addition, fat mass was positively associated to bone maturation in spine in girls, this may be mediated by increasing synthesis of estrogen in the adipose tissue [31] but also, recent studies have focused on hormones that link fat to bone. Adipokines, such as leptin, modulate bone cells through both direct and indirect actions, and overall, most studies indicate a positive relationship between leptin and bone mass [32,33,34,35]. In accordance with these findings, our data showed a positive association between %BF and total body BMC, independent of CRF fitness.

### CRF and BMC

CRF has been directly associated with bone mass in adolescents [36] by the relationship with muscle mass [37]. However Kemper H.C. showed that in adolescents and the young, only neuromotor fitness, as defined by muscular fitness and speed, and not cardiorespiratory fitness was related to bone mineral density [38]. In our study, children with good CRF had less total body BMC after controlling for age and sex (model 1) than their peers with lower fitness levels. However, once we controlled for VPA and %BF (model 3), children with good CRF had more total body BMC than their peers with lower fitness, suggesting that previous differences observed from model 1 are due to differences in VPA and %BF between CRF groups.

### VPA and BMC

The literature consistently identifies physical activity as one of the most important determinants of BMC in children and adolescents. Habitual VPA [12,39,40], through changes in lean mass [41], has been positively associated with cortical bone mass [39]. Our data showed that higher levels of VPA were inversely related to a healthier bone status in children. This might be explained by the role of percent body fat as VPA is negatively related to body fatness [42], and therefore, children in the higher VPA category are frequently lighter and as a consequence have a lower BMC [26].

### Percent body fat and CRF as mediators between VPA and BMC

There is consistent evidence regarding the bivariate association of BMC with both fat mass [14,15,43] and CRF [3]. Likewise, the relationship between PA and total body BMC in children has been extensively established [6,44,45]. However, although PA has been considered as a determinant of bone mass, it has not been fully clarified whether %BF and CRF act as confounders or as mediators. Our study confirms the crude relationships between %BF and CRF with total body BMC and clarifies the mediating role of %BF and CRF in the relationship between VPA and total body BMC. To date, it has not been clear whether %BF and CRF could jointly act as mediators in the relationship between VPA and total body BMC. Our data supports that %BF and CRF have a great influence on the relationship between VPA and total body BMC and, more specifically, that the relationship of CRF levels on bone mass is fully mediated by %BF and that VPA was not modified the %BF.

The current study has several limitations that should be acknowledged. Firstly, the cross-sectional design prevented us from making cause–effect inferences. Secondly, sexual maturation was not included as a covariate in our analyses since it was only available in a reduced proportion of the whole sample. However, all the children for whom the sexual maturation data were obtained were in Tanner stages I-II, and therefore the sample could be considered homogeneous regarding this variable. Thirdly, we may have underestimated VPA as the accelerometers were set at 60-s epochs and therefore, short bouts of VPA may have not been recorded [46]. Finally, the relationships analysed here are probably influenced by more than two mediator variables; future studies using structural equation procedures might be useful in clarifying more specifically the potential mediator role of each factor.

## Conclusions

Our findings are important from a clinical and public health point of view because they show that %BF and CRF influence the relationship between VPA and bone mass in children. Moreover it should be noted that the influence of %BF on bone mass is stronger than that of CRF, and also that %BF mediates the association between CRF and bone mass in children.

## Supporting Information

S1 Fig Simple mediation models of the relationship of vigorous physical activity (VPA) and cardiorespiratory fitness (CRF) with bone mineral content (BMC), using lean mass (LM) as mediator, controlling for age. **p< 0.01 *p< 0.05
(PPTX)

S2 Fig Multiple mediation analyses of the association between vigorous physical activity (VPA) and total body bone mineral content (BMC), with cardiorespiratory fitness (CRF) and lean mass (LM) as mediators, controlling for age. (DOCX)

S1 Table ANCOVA models comparing means of total body bone mineral content (BMC) by total lean mass (TLM), cardiorespiratory fitness (CRF) and vigorous physical activity (VPA) categories in children. (DOCX)
