# Supplementary material for: Physical Activity and Bone Health in Schoolchildren: The Mediating Role of Fitness and Body Fat
Source: PLoS One. 2015 Apr 27;10(4):e0123797. doi: 10.1371/journal.pone.0123797 (PMC4411135; doi:10.1371/journal.pone.0123797)
Supplement: S1 Fig — **p< 0.01 *p< 0.05 (PPTX) [file pone.0123797.s001.pptx]

## Slide 1
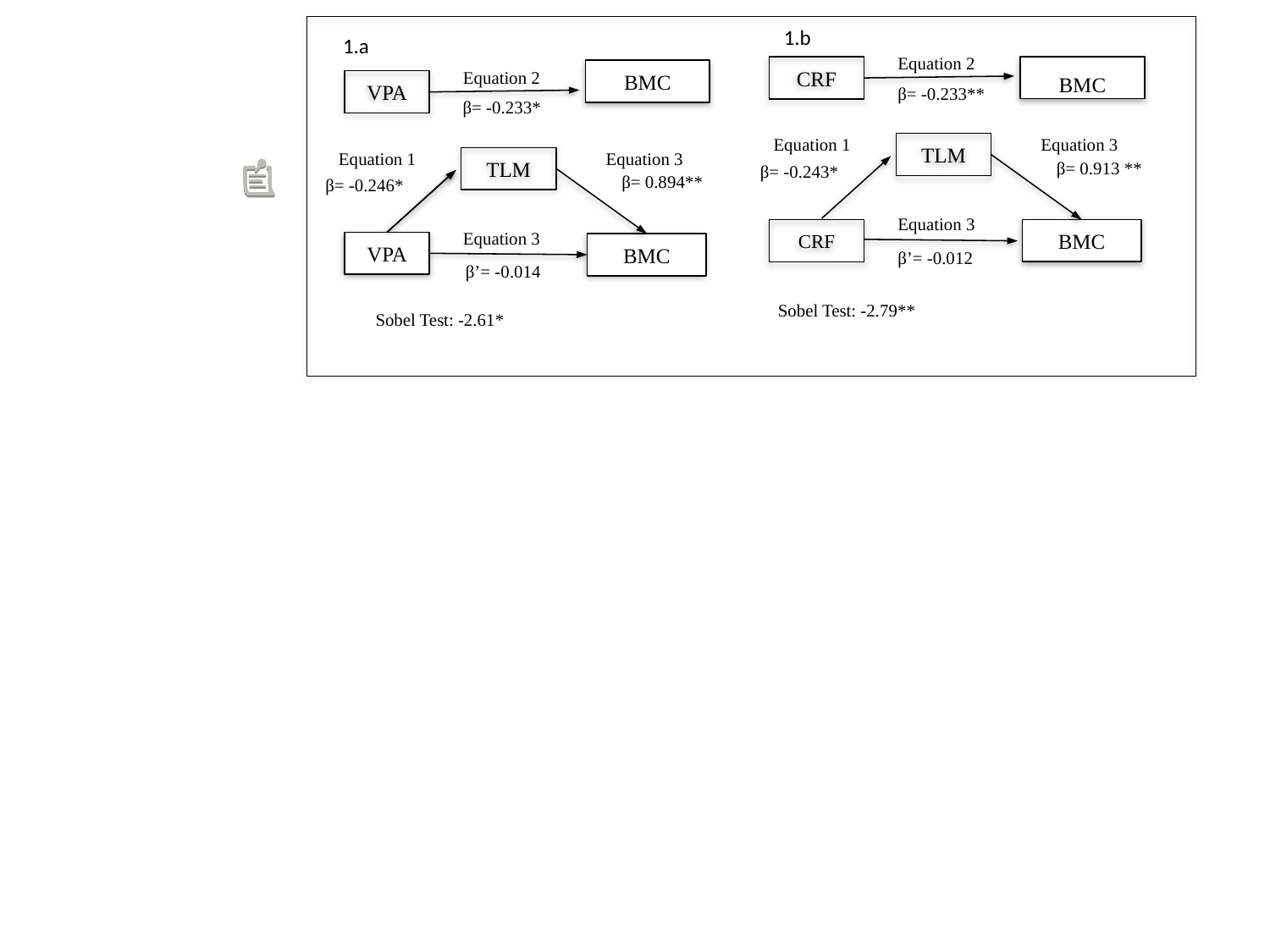

1.b
1.a
Equation 2
CRF
BMC
β= -0.233**
Equation 1
Equation 3
TLM
β= 0.913 **
β= -0.243*
Equation 3
BMC
CRF
β’= -0.012
Sobel Test: -2.79**
Equation 2
BMC
VPA
β= -0.233*
Equation 1
Equation 3
TLM
β= 0.894**
β= -0.246*
Equation 3
VPA
BMC
β’= -0.014
Sobel Test: -2.61*
