# Supplementary material for: Physical Activity and Bone Health in Schoolchildren: The Mediating Role of Fitness and Body Fat
Source: PLoS One. 2015 Apr 27;10(4):e0123797. doi: 10.1371/journal.pone.0123797 (PMC4411135; doi:10.1371/journal.pone.0123797)
Supplement: S2 Fig — (DOCX) [file pone.0123797.s002.docx]

|  | **Coefficient** | **s.e.** | **p** | **Bootstrap 95%CI** |
| --- | --- | --- | --- | --- |
| Total effect (c Path) | -4.979 | 2.252 | 0.029 | (-9.442; -0.515) |
| Direct effect (c’Path) | -0.461 | 1.401 | 0.743 | (-3.238; 2.317) |
| Indirect effect (via mediators) | -4.518 | 1.987 |  | (-8.370; - 0.498) |
| VPA→CRF→BMC | -0.391 | 0,791 |  | (-1.838; 1.284) |
| VPA→CRF→TLM→BMC  VPA→ TLM→BMC | -2.351  -1.777 | 1.353  2.190 |  | (-5.332; -0.030)  (-5.920; 2.724) |

d: -380.543 (208.940)

VPA

BMC

CRF

a_1_:0.137

(0.019)**

b_1_: -2.847

(5.785)

TLM

a_2_:-39.505

(51.227)

b_2_= 0.045

(0.003)**

c’: -0.461(1.401)

----------------------

c: -4.979 (2.252)*

Values are coefficient (s.e.)

* p< 0.05 **p< 0.001
