# Supplementary material for: Physical Activity and Bone Health in Schoolchildren: The Mediating Role of Fitness and Body Fat
Source: PLoS One. 2015 Apr 27;10(4):e0123797. doi: 10.1371/journal.pone.0123797 (PMC4411135; doi:10.1371/journal.pone.0123797)
Supplement: S1 Table — (DOCX) [file pone.0123797.s003.docx]

| **Total body BMC (g)** | | | | | | | | | | | | |
| --- | --- | --- | --- | --- | --- | --- | --- | --- | --- | --- | --- | --- |
|  | **Total lean mass** | | | | **Cardiorespiratory fitness** | | | | **Vigorous physical activity** | | | |
|  | Low  n =33 | Medium  n =66 | High  n=33 | p | Poor  n = 38 | Satisfactory  n=60 | Good  n=24 | p | Poor  n = 32 | Satisfactory  n=55 | Good  n=25 | p |
| Model 1 | 1203.83±26.66 | 1360.82±19.181 | 1504.27±26.41^a^ | **<0.001** | 1451.20±29.11^a,b^ | 1326.04±24.23 | 1302.92±33.35 | **0.001** | 1405.32±32.59^a^ | 1330.60±23.62 | 1286.91±35.85 | **0.046** |
| Model 2 | 1255.52±34.57 | 1347.95±24.44 | 1438.75±34.88^b,c^ | **0.003** | 1399.63±33.81 | 1323.72±27.07 | 1306.38±37.88 | 0.147 | 1386.20±33.08 | 1325.92±23.24 | 1323.68±38.78 | 0.301 |
| Model 3 | 1259.44±38.55 | 1347.86±24.56 | 1434.46±39.52 ^c^ | **0.025** | 1321.81±38.25 | 1332.35±25.66 | 1369.76±39.66 | 0.695 | 1362.57±32.23 | 1329.19±22.16 | 1346.40±37.50 | 0.675 |
